# Synthesis, Characterization, and Application of 1-D Cerium Oxide Nanomaterials: A Review

**DOI:** 10.3390/ijms11093226

**Published:** 2010-09-13

**Authors:** Kuen-Song Lin, Sujan Chowdhury

**Affiliations:** Department of Chemical Engineering and Materials Science/Fuel Cell Center, Yuan Ze University, Chung-Li City, Taiwan

**Keywords:** cerium oxide, nanotube, nanomaterials, one dimensional nanostructure, formation mechanism

## Abstract

The present work provides a comprehensive overview of the recent progress of research work toward developing new one dimensional (1-D) ceria (CeO_2_) nanomaterials. The review has been classified into three parts: the preparation procedures with identification of the existing different dimensional ceria nanomaterials, the formation mechanisms, and an analysis of their applications. From literature survey, it is inaugurated that the fundamental structures of the ceria nanomaterials constructively dominate their properties and applications. In addition, this work will also provide a perspective on the future technical trends for the development of different dimensional CeO_2_ nanomaterials.

## 1. Introduction

In the recent years, development of lanthanide compounds (Z = 57–71) such as ceria nanomaterials have been paid much attention. Nanoscale ceria materials can rapidly formed redox Ce^4+^/Ce^3+^ sites into their 4f shell of ions; assisting industrial applications [1–3]. Since such small dimensions possess specific surface areas and have excellent fundamental technological consequences, the preparations of one dimensional (1-D) nanostructures are widely attractive. Fabrication of nanocrystalline with the desired dimension and shape to provide effective activity and efficiencies for catalytic purposes is still an ultimate challenge in modern material research. In the past few years, well-defined 1-D ceria nanostructures with various morphologies such as nanorods, nanowires, nanotubes, nanopolyhedrons, *etc*., have been successfully fabricated by a variety of methods [1–15].

Nanoscale zero-dimensional ceria clusters could effectively provide individuality and have led to substantial advances in particle size, including nanoscale catalysis. In addition, nanoscale ceria materials of 1-D structure are represented with surface morphologies, allowing attractive applications for catalytic reactions. Moreover, ceria nanomaterial research has focused on the scheme of the physical treatment, based on the controlling of the reaction time, temperature, pressure *etc*. In this perspective, preparations of ceria nanomaterials generally fall into four basic steps: synthesis of precursors, treatment of precursors before conversion to oxides, conversion of precursors to mixed oxides, and post treatment of mixed oxide material. These methods have been used for preparing not only pure ceria but also for doped and mixed ceria nanomaterials. Some of these methods are included with the precipitation, sol-gel, thermo-decomposition, *etc*. Several ways have also been used in colloidal systems to obtain ceria nanomaterial, such as emulsion and microemulsion. On the other hand, in the presence of surfactants and polymers aiming to enhance physical or chemical properties such as surface area, sintering resistance, activity towards a certain reaction, *etc*, there is a particular focus to synthesized different 1-D nanomaterial to amplify their potential applications, which are covered in this review. This typical procedure for preparation and characterization of the surface properties of 1-D ceria nanomaterials has been adversely associated with the preparation mechanism and this is also described in this review.

## 2. Recent Works on the Preparation of 1-D CeO_2_ Nanostructures

Ceria is associated with rich oxygen vacancies and higher redox ability between Ce^3+^ and Ce^4+^, therefore promoted as a higher capacity oxygen storage material. In addition, 1-D cerium oxide nanorod, nanowire or nanotube (Ce–NT) draw attention due to their novel properties for the fabrication of nanodevices and unique atomic efficiencies with rapid response to changing condition in the catalytic system.

### 2.1. 1-D CeO_2_ Nanorod

The solvent composition, surfactant, and the cerium source precursor are of importance in the final product morphology [1–5]. The reaction temperature, concentration of the cerium precursor, and reaction time have significant influence on the yield of CeO_2_ nanorods [1,2]. According to Ho *et al*. [2], ethylene glycol-mediated synthesis had been widely used owing to the impact of three significant physical properties: (1) a high dielectric constant, which enhances the solubility of inorganic salts; (2) a high boiling point (195 °C at atmospheric pressure), which makes it possible to carry out the preparation of inorganic compounds at relatively high temperatures; (3) its strong reducing power. Additionally, Ho *et al.* also observed that a higher precursor concentration with lower reaction time provides spherical shaped cerium oxide and increasing the reaction time consequently extended the spherical shape into 1-D rod structures [2]. In-addition, with similar experimental conditions and a lower precursor concentration, they obtained the spindle shape nanostructure. On the other hand, Thang and coworkers [3] successfully obtained needle shaped nanostructures at an environment with higher amount of oxidizing agent and a higher concentration of the precursor. The surface area of the 1-D cerium oxide was increased significantly with the calcination, attributable to the higher temperature treatment initiating the crystallization into the nanostructures. Surfactant plays an effective role for the preparation of 1-D nanophase compounds and has been adversely observed in the past decades [1–11]. Vantomme *et al*. [4] and Pan *et al*. [5] reported the formation of CeO_2_ nanorods with a diameter of 10–25 nm at 80–160 °C by the presence of cetyltrimethylammonium bromide (CTAB). Pan and coworkers [5] also synthesized the CeO_2_ nanoplates by hydrothermal reactions with CTAB. They controlled the conversion of nanoplates into nanotubes and nanorods by using changing CTAB/Ce^3+^ ratio values, reaction time, and temperature. Similarly, Zhou *et al*. [6] obtained the CeO_2_ nanorods of 15–30 nm in diameter and lengths of up to tens of micrometers by a precipitation method combined with the hydrothermal treatment.

Later on, Huang *et al*. [7] synthesized Au/CeO_2_ nanorods with the wet chemical reducing system in the presence of NaBH_4_ solution as a reducing agent. They also observed that hydrothermal temperatures influenced the nucleation and crystal growth of the CeO_2_ nanorod. Morphological transformation of the nanorod was not completed with hydrothermal temperatures below 150 °C at 5 or 10 M KOH solution. Consequently, higher alkaline concentration provides thicker nanorod structures. Therefore, it would be considered that higher alkaline concentration is involved in increasing the width of the nanostructures rather than the nucleation of length of the samples. Similar to this approach, it was also confirmed for the formation of different shape of ceria oxide 1-D nanostructures with the presence of different concentrations of alkali [8]. At lower precipitation concentrations, the shape of nanopolyhedra, and at higher concentrations, a mixture of rod and polyhedral shapes were provided, respectively. On the other hand, the precipitant mainly formed the cubic and rod shape structure at higher temperature and higher concentration, respectively.

The one-step synthesis of CeO_2_ nanorods is still a challenge. In this case, ultrasonication methods have been successfully used to prepare nanorods. In the previous reports, the synthesis methods of CeO_2_ nanorods were relatively complicated and always needed high-temperature, high-pressure or long-time treatments [9–12]. In addition, Qi *et al*. [9] synthesized the thicker CeO_2_ microrod (200–250 nm in diameter and 600–1200 nm in length) by an ultrasonication process then surfactant assisted hydrothermal method. Furthermore, Zhang *et al*. [11] prepared 1-D ceria nanorods at room temperature in a one-step process through polyethylene glycol (PEG) surfactant and alkali solution. They confirmed that vigorous agitation without ultrasound at various temperatures (25, 40, and 60 °C) would form only nanoparticles as the sole products, even with a longer reaction time. Moreover, the concentration of the surfactant (e.g., PEG or CTAB) significantly affects the formation of 1-D nanorods [4,5,11]. Recently, Feng *et al*. [12] approached the microwave-hydrothermal method for the facile, rapid synthesis of higher yields of 1-D CeO_2_ with average sizes of ~1.6 nm to ~20 nm. Compared with a conventional hydrothermal method, the microwave-assisted hydrothermal method shows advantages of rapidity, convenience, cost-effectiveness and could be potentially extended to the synthesis of other nanoparticles and nanorods.

**Figure 1:**
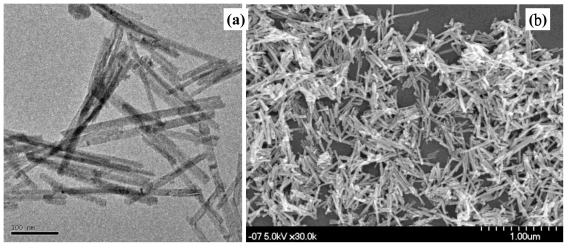
(**a**) Transmission electron microscope (TEM) and (**b**) scanning electron microscope (SEM) images of ceria nanorods synthesized using a hydrothermal method.

Recently, we have successively developed the CeO_2_ nanorod at a higher concentration of alkali (e.g., NaOH) solution and without surfactant with the well known hydrothermal method at 100 °C for 24 h. Morphology of the CeO_2_ nanorod is identified in the low-magnification transmission electron microscope (TEM) and field-emission scanning electron microscope (FE-SEM) images of Figure 1(a),(b), respectively. It was recognized that the 1-D CeO_2_ nanorod have a diameter of 20–40 nm and a length of 200–300 nm. Nanorod structure consisted of fluorite structure were confirmed with X-ray Diffraction (XRD) patterns after drying at 60 °C overnight and calcined at 300 °C for 3 h in the presence of air.

### 2.2. 1-D CeO_2_ Nanowire/Nanofiber

**Figure 2:**
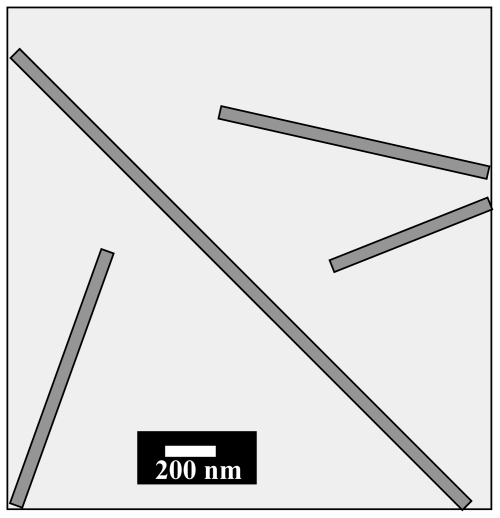
A schematic image of CeO_2_ nanowires formed by using anodic alumina membranes (AAM) as templates.

Surfactants were frequently used for the fabrication of cerium oxide 1-D nanowire/nanofibers. Qizheng *et al*. [13] were the first to demonstrate the electrospinning technique for the formation of PVP/Ce(NO_3_)_3_ composite fibers. They fabricated the cerium oxide hollow nanofibers with calcining the composite fibers at 600–800 °C for 10 h. According to the FE-SEM microphotographs, the diameters of CeO_2_ hollow nanofibers (300 nm at 600 °C and 600 nm at 800 °C, respectively) were smaller than those of PVP/Ce(NO_3_)_3_ composite fibers (1–2 μm), with the length of greater than 50 μm. They observed, through TG-DTA and FTIR data analysis, that the calcination temperatures largely influenced the formation of CeO_2_ hollow nanofibers. In a typical procedure, Gu *et al*. [14] successfully synthesized mesoporous ceria nanofibers, nanobelts, and rodlike nanoparticles using a reverse micelle method. In addition, BET surface area and pore volume of the nanobelts (114.9 m^2^g^−1^ and 0.1470 cm^3^ g^−1^, respectively), were about twice as high as those of the nanofibers (54.41 m^2^g^−1^ and 0.09051 cm^3^ g^−1^, respectively). On the other hand, Tang *et al*. [3] simply used the hydrothermal method to achieve nanowires without the presence of surfactant. They observed that the presence of acidic precipitant H_2_O_2_ with 0.1 M Ce(NO_3_)_3_ produces the nanowire and nanocubes, whereas lower concentration of the precursor (0.05 M Ce(NO_3_)_3_) formed only nanowire diameters of 20–70 nm and lengths up to 40 μm in the hydrothermal process at 250 °C for 3 h. Furthermore, aggregated nanoneedles have been formed when the oxidizing agent H_2_O_2_ was absence and thus act as a template agent in this experiment. Nanowires were structurally uniform and single crystalline. The interplane distance in this research was obtained as 0.28 nm, corresponding to the separation between the (200) lattice planes of cubic CeO_2_. The ordered CeO_2_ nanowire arrays embedded in anodic alumina membranes (AAM) fabrication are also a novel technique. La *et al*. [15] and Wu *et al*. [16] fabricated CeO_2_ nanowires with a diameter of 60–70 nm by using AAM as templates. As showed in Figure 2, anions and cations are conversely migrated into the hexagonally ordered nanochannels of the AAM and are reacted inside the channels to form 1-D nanostructures.

Sun *et al*. [17] synthesized CeO_2_ nanowires, 30–120 nm in diameter, by a precipitation method combined with thermostatic treatment using sodium bis(2-ethylhexy) sulfosuccinate (AOT) as a template. By using a similar method, Yan *et al*. [18] and Vantomme *et al*. [4] carried out the ceria nanowire preparation with the presence of easily available CTAB. Yada *et al*. [19] prepared different types of 1-D nanowire structures with the presence of different order alcohol and AOT as anions at 700 °C or above. In the presence of AOT, adding lower order alcohol such as alkyl or butyl alcohol and higher order alcohol (octyl or dodecyl alcohol) only produced nanowire and the nanoring shape nanowire (diameter of ~280 nm and width of ~80 nm), respectively. In a typical reverse micelles procedure, Gu *et al*. [14] successfully synthesized mesoporous ceria nanofibers at the lower aging temperature at 30 °C with a diameter of 50–200 nm and length of more than 50 μm with the presence of nonionic surfactant Triton X-100. On the other hand, nanobelts materials with length of a few tens of μm, widths ranging from 0.5 to 5 μm, and the thicknesses ranging from 20 to 100 nm have been prepared at the slightly higher aging temperature at 40 °C and constant time of 48 h. In addition, Yang and Guo [20] also employed octadcylamine (C_18_H_37_NH_2_) (cationic surfactants) as the structure–directing agent to synthesize CeO_2_ nanowires with a diameter of 10–25 nm. Tuning the ammonium acetate concentration through the precipitation method, Bugayeva *et al*. [21] controlled the particle size, shape, and agglomeration of the 1-D nanowire. The hydrated CeO_2_ nanowires as thin as 5 nm in diameter and nanoneedles with various aspect ratios were obtained via a chemical precipitation technique in the presence of ammonium acetate.

### 2.3. 1-D CeO_2_ Nanotube

Generally, the tubular structure itself may consist of higher thermal, chemical, and structural stability [22–26]. Various preparation conditions have been employed to synthesize 1-D Ce-NT materials, such as the use of different surfactants and templates, ultrasound treatment, hydrothermal method with different temperatures, aging effect, and acidic treatment. The template synthesis method is an effective way for preparation of the nanomaterials in the presence of polymeric filtration membrane and similar materials [22–25]. Yang *et al*. [23] also synthesized the fluorite-type Ce-NT with an outer diameter of 10–20 nm and inner diameter of 5–6 nm. Ce(OH)CO_3_ was attained by a hydrothermal method using Ce(NH_4_)_2_(NO_3_)_6_ as the Ce source, octadecylamine as a surfactant template, and urea as a precipitation agent. In addition, a higher temperature and higher concentration of CTAB as a surfactant were used for the synthesis of Ce-NT in the two-step procedure by Pan and coworkers [5]. In the first step, a higher concentration of the CTAB led to an increase in the absorption force between the CTA^+^ and Ce^3+^/Ce^4+^ ion pairs and accelerated the formation of lamellar sheet. In the second steps, Ce-NT was formed as a result of rolling up the lamellar sheets. Chen *et al*. [24] studied three different ways for the formation of ceria nanotube on the basis of the Kirkendall effect (denoted as K-type), Template (T-type), and lamellar rolling (L-type). The K-type Ce-NT had been prepared by congregating Kirkendall voids and subsequent calcinations were acquired in the presence of air at 600 °C for 4 h. In addition, T-type and L-type nanotubes had been obtained without any calcination. Precipitant and the reaction temperature are implicated in the formation of the K-type ceria nanotube.

**Figure 3:**
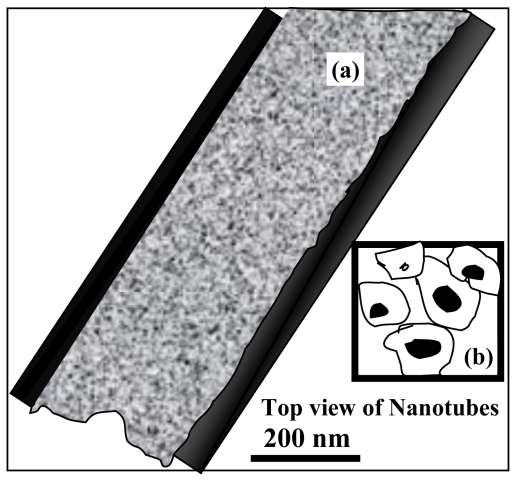
(**a**) Schematic microstructure of Ce-NT and (**b**) insert represented as the top-view of Ce-NT.

Carbon nanotube (CNT) as a template plays a significant role in the formation of 1-D ceria nanostructures. It was reported that the surface of the template was covered with ceria nanomaterials and possesses Ce-NT. In addition, higher temperature treatment was carried out for the removal of the templates [25–28]. The CNTs were refluxed in a mixture of concentrated KOH and NaOH at 450 °C and that could be coated with CeO_2_ for the formation of 1-D nanotubular structures [27,28]. The formation of Ce-NT is assisted with different methods just like ultrasonication, facile solvothermal method [25,26,29–31]. The preparation of Ce-NT is composed of several tiny interconnected nanocrystallites of about 10 nm in size. The pretreatment of CNTs and calcination temperature have been considered as crucial factors for determining the formation of Ce-NT. Metal ion doping is a promising technique to control the properties of material. Doping of metallic ion on the nanomaterials can influencethe surface morphology, nanocrystal shape, and growth in solution. Fuentes *et al*. [22] obtained the mixed Zr-Ce-NT oxide in the presence of polycarbonate film as a template through the microwave radiation at 800 W. Furthermore, Lu *et al*. [32] reported a route for the synthesis of Ce-NT within an AAM template (Figure 3).

It is evident that complete and controlled conversion of CeO_2_ nanostructures through templates is not readily achievable. Additionally, fabrication and removal of the template have been achieved as very troublesome techniques for the Ce-NT synthesis process. Therefore, the formation of 1-D nanotubes with the absence of templates has been attractive owing to simple, quick, and economical considerations. On an important low-cost basis, Miao *et al*. [33] developed the procedure of ultrasound irradiations, in order to prepare Ce-NT from ceria nanoparticles at room temperature. In addition, Santos *et al*. [34] explained that the calcined temperature readily affected the crystallinity and morphology of the CeO_2_ nanostructure. Thus, the development of a facile and controllable formation of Ce-NT with proper crystalline structure is of great significance.

One of the most notable characterizations of the fluorite Ce-NT has been recently developed by a hydrothermal method. Han *et al*. [35] synthesized the yellowish CeO_2−_*_x_* nanotubes, nanowire, and nanoparticles in two steps. At the beginning, the samples were prepared at 100 °C in the presence of 7 mL of 5% ammonia hydroxide solution and then aged at 0 °C for 45 days. This procedure is time consuming. Tang *et al*. [36] proposed the lamellar rolling of the Ce(OH)_3_ crystal nanotubes through the alkali treatment of the trivalent ceria salt CeCl_3_ at 120 °C under an oxygen free environment with the hydrothermal method. They observed that 1-D Ce-NT was obtained from the annealing of Ce(OH)_3_ crystal nanotubes in the reducing atmosphere.

It was reported by Pan and coworkers [37] that cerium oxide nanorods are easily obtained under alkali treatment at room temperature. In addition, they explained that accumulation of the Ce^3+^ ion for 72 h on the cerium oxide nanorod surface would provide Ce-NT in the hydrothermal condition around 100 °C. As well, at increasing temperature the deposition of Ce^3+^ ion occurred at the tip of the nanorod and formed the nanowire and subsequently nanocubes. It was also shown that a larger surface area was achieved by the lower temperature nanorod preparation. However, this method is an effective way for the preparation of Ce-NT in the case of the template free controlled conversion system. Chen *et al.* [38] synthesized Ce-NT with a simple solid liquid interface reaction route in the absence of any surfactants by employing Ce(OH)CO_3_ nanorods as precursors.

As a synthesis of hydrothermal method, Zhou *et al.* [39] converted CeO_2_ nanorods into nanotubes in an acidic treatment like H_2_O_2_ solution assisted by ultrasonication. The converted Ce-NT has higher reducible property, which was due to the higher activity of CeO_2_ surface (100) than that of common surface (111) [39,40]. In addition, CeO_2_ nanorods consisted of Ce^4+^ as a surface material and Ce^3+^ as inside [39]. On the other side, Han *et al*. [35] obtained the opposite phenomenon, since the fraction of Ce^3+^ is significantly larger than that of CeO_2−_*_x_* nanoparticles with the same diameter. Thus, Ce^3+^ ions remained on the surface of the 1-D Ce-NT. Chen and coworkers [41], through the Kirkendall effect, obtained 1-D Ce-NT in which Zr^4+^ ions may act as the catalyst to promote the diffusion rate of Ce^3+^/Ce^4+^ ions inside the nanorod. According to a partial oxidation of Ce^3+^ ions and differential rate of diffusion between Ce^4+^ and Ce^3+^ ions inside the material, the metal hydroxide nanorods gradually decompose to form Zr*_x_*Ce_1−_*_x_*O_2_ nanotubes. Furthermore, Martin *et al*. [42] used the atomistic simulation techniques based on the Born model of solids to observe multilayer Ce-NT with a wall thickness of 5.5 nm and a lumen diameter of 4.8 nm. Besides, the 1-D ceria nanostructure was achieved with the electrochemically-synthesized route through change of the electric field, strength, and direction by Fu *et al*. [43]. They acquired the morphologies of ceria nanomaterials from nanoparticles and nanorods to nanowire by simply changing the potential direction and time of anodic oxidation.

### 2.4. Other Types of 1-D CeO_2_ Nanostructure

Reverse micelles provide spontaneous self-assemble of surfactants in solution for the formation of nanorods. Kuiry *et al*. [44] reported that the cylindrical supra-aggregates and their subsequent growth occurred by preferential assembling of ceria nanorods along the longitudinal direction with the addition of AOT/toluene/water and H_2_O_2_/AOT/toluene/water microemulsions after a few weeks of aging. Such nanorods have an aspect ratio of 6 with a diameter of approximately 40 nm. In addition, according to the TEM analysis, it was proposed that the abrupt change in surface free energy in the micelle might form the cone-shaped portions at both ends of the nanorods. However, Tang *et al*. [3] proposed and explained that the concentration of an oxidant such as H_2_O_2_ would significantly affect to impose the cone type or needle like phenomenon in the 1-D cerium structure. On the other hand, Ge *et al*. [45] successfully used the emulsion liquid membrane system to synthesize CeO_2_ sponge-like rods with diameters of 170–810 nm and lengths of 5–10 μm, which were successfully fabricated through a route of liquid emulsion membrane followed by heat treatment.

## 3. Formation Mechanism of 1-D CeO_2_ Nanostructures

**Scheme 1:**
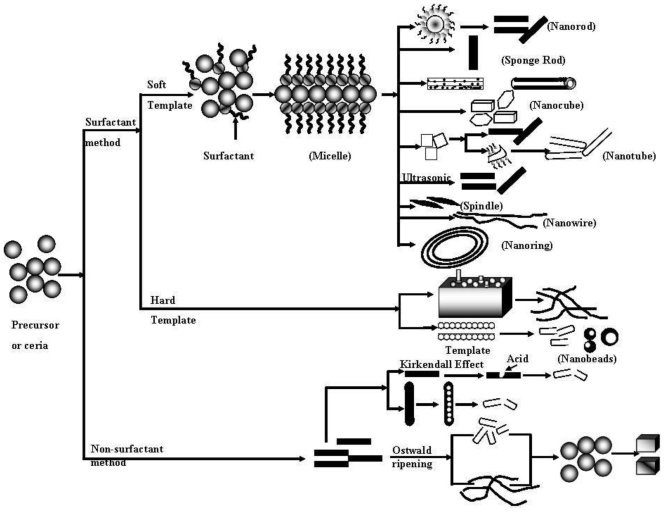
Details of the reaction mechanism pathways for the formation of ceria nanostructures.

In recent years, literature data on the kinetics of 1-D ceria nanostructure has become plentiful. The preparation of the 1-D Ce-NT, nanorod or nanowire was thoroughly investigated by the surfactantassisted preparation and non-surfactant assisted Kirkendall coarsening; also known as the Ostwald ripening method as shown in Scheme 1. At the beginning, a famous paper by Terribile *et al*. [46], reported the first complete reaction mechanism of surfactant assisted 1-D Ce-NT nanostructure, and this is in essence still regarded to be valid. Afterwards, several fundamental preparation mechanisms assisted to obtain 1-D ceria nanostructure.

### 3.1. Surfactant Assisted 1-D CeO_2_ Nanostructure Formation

Surfactant plays an important role in the preparation of ceria nanostructure. The reaction of cerium salts (either chloride or nitrate) under basic conditions with ammonia at room temperature results in the precipitation of gelatinous, hydrous cerium oxide. If the reaction is conducted in the presence of the “soft template” as cationic surfactants (*i.e.*, alkyltrimethylammonium salts, CTAB, octadcylamine or ethylenediamine (C_2_H_4_(NH_2_)_2_)), hydrous cerium oxide can incorporate the organic molecule by exchange with surface OH^−^ groups. This approach follows the observation that hydrous oxides can exchange either cations or anions, depending on the pH of the medium [19,46]. If the pH is higher than that of the isoelectric point of hydrous cerium oxide (6.75–8, depending on the environment) then incorporation of cationic surfactants takes place. The size and shape of the 1-D nanostructure is greatly influenced through the reaction time, reaction temperature and surfactant/Ce^3+^ ratio in the initial solution [5,17–23,46–47].

Scheme 1 shows the possible formation mechanism of CeO_2_ with different morphologies [5], in where surfactant (CTS^+^) is firstly absorbed on the surface of CeO_2_ nanoparticles (Equation (1)). The absorbed ligand molecules in the equation are likely to interact preferentially with the (111) surface plane to (100) at lower temperatures [4,40]. As pointed out here, Terribile *et al*. reported the first complete reaction scheme for 1-D ceria preparation in 1998 [46].

(1) $$CeOH↔{CeO}^{-}+H^{+}↔{CeO}^{-}CTS^{+}$$

According to Terribile *et al*., the soluble isolated Ce^3+^ under basic conditions oxidizes to a hydrated Ce^4+^ formulated as Ce(H_2_O)_x_ (OH)_y_ ^(4−y)+^ (Equation (2)), which then readily combines with the surfactant in accordance with reaction equation (3). This step can also be viewed as the two individual steps for the formation of polymeric hydrous oxide, which then reacts with the alkylammonium salt (Equations (3a) and (3b)) at a pH value well above that of the isoelectric point of ceria. Under these conditions, surfactant and the deprotonated hydroxy group form an inorganic/organic composite, which upon drying and calcination (Equations (4) and (5)) originates pure mesoporous cerium oxide with high surface area. According to reaction (3), they observed that the surfactant is able to promote oxidation of Ce^3+^ to Ce^4+^ and formation of hydrous oxide in solution, before drying. The presence of more surface Ce^4+^ atoms is a consequence of the smaller particles formed with the surfactants with a higher number of exposed Ce^4+^ atom.

(2) $$2{Ce}^{3+}+\frac{1}{2}O_{2}\overset{H_{2}O}{↔}2{Ce\left(H_{2}O\right)}_{x}{\left(OH\right)}_{y}^{(4-y)+}$$

(3a) $$n{Ce\left(H_{2}O\right)}_{x}{\left(OH\right)}_{y}^{(4-y)+}↔{\left(-CeHOHOCeHO\;H\right)}_{n}.{mH}_{2}O+H_{2}O$$

(3b) $${\left(-CeHOHOCeHO\;H\right)}_{n}.{mH}_{2}O+surf↔nCeOx\;{\left(O-surf\right)}_{y}.{mH}_{2}O$$

(4) $$nCeOx{\left(O-surf\right)}_{y}.{mH}_{2}O\overset{drying}{\to }nCeOx\;{\left(O-surf\right)}_{y}+{mH}_{2}O$$

(5) $$CeOx{\left(O-surf\right)}_{y}\overset{Calcinatio\;n}{\to }{CeO}_{2}+org+CO_{2}$$

$$O-surf=ONC_{19}H_{42}$$

Therefore, surfactant quantity easily affects the formation of the 1-D nanostructures and lower content of surfactants have been readily involved to produce smaller quantity of nanorods with a shorter length in size [4,18,20,40]. In addition, the exposed surfaces from the combination of the surfactant ceria surfaces tend to reduce the surface energy to form a cubic plane structure. A similar phenomenon for the formation of 1-D ceria nanomaterials was explained by Pan *et al*. [5] in 2008 (Scheme 1). They observed that the lower capping of the CTS^+^ forms the thicker ceria nanoplates and thus partially converts into nanorods. Furthermore, they considered a rolling strategy for the nanoplates to obtain nanotubes at higher capping of CTS^+^. The Cerium hydroxide, which combined by the hydrated Ce^4+^ ions with H_2_O molecules or OH^−^ ions, polymerized at the micelles-solution interface and formed the nanowire structure clusters [20].

The distribution of the surfactant and template both significantly played an important role in the formation of nanowire and nanorod. According to Kuiry *et al*. [44], precursor is formed into nanoparticles by the process of nucleation through surfactant and growth occurs inside the cores of the micelles (Scheme 1). Hydroxyl ions are formed locally inside each micelle core as an intermittent product of the dissociation of hydrogen peroxide during the interaction (Equations (6) and (7)) [44].

(6) $${Ce}^{3+}+OH^{-}+\frac{1}{2}H_{2}O_{2}\to Ce{\left(OH\right)}_{2}^{2+}$$

(7) $$Ce{\left(OH\right)}_{2}^{2+}+2OH^{-}\to {CeO}_{2}+2H_{2}O$$

The occurrence of this process is confirmed by color changes (colorless, violet, brown or yellow) during the reaction, which is consistent with a progressive variation of the redox state of cerium. The violet color is characteristic of poorly defined mixed valence hydrates intermediates, which finally give yellow Ce(IV) oxide. In the absence of surfactant, complete oxidation to Ce^4+^ occurs only at the drying stage when the precipitate turns yellow [46]. In addition, change of surface free energy in the micelle is readily accommodated to obtain the spindle shape nanostructures. The concentration of the oxidant such as H_2_O_2_ would have a significant effect to impose the cone type or needle like phenomenon in the 1-D cerium structures [2,3]. Dengsong *et al*. [11] explained the ultrasonication method for the formation of the ceria nanorod. In the presence of air and alkaline solution, Ce^3+^ oxidation state is unstable compared with the Ce^4+^ oxidation state, thus resulting in the formation of hydrated Ce^4+^ oxide (Equation (8)). Later on, CeO_2_ nanoparticles readily fuse with OH^−^ ions through PEG. In addition, with the ultrasonication, the generated bubbles asymmetrically increase the collision between adjacent PEG adsorbed nanoparticles and as a result form the 1-D ceria nanorod in reaction Equation (9). It is well known that the strongly adsorbed stabilizer prevents the aggregation between colloidal nanoparticles due to its steric hindrance effect.

(8) $${Ce}^{3+}+3OH^{-}+\frac{1}{4}O_{2}(air)+\left(n-\frac{3}{2}\right)H_{2}O\overset{Ultrasonication}{\to }{CeO}_{2}.nH_{2}O$$

(9) $${CeO}_{2}.nH_{2}O\overset{Ultrasonication}{\to }{CeO}_{2}+nH_{2}O$$

Recently, AAM (hard template) was fabricated by a novel technique in which anions and cations conversely migrated into the hexagonally ordered nanochannels of the AAM and reacted inside the channels to form 1-D nanostructures [15,16]. In the presence of a basic environment, Ce^3+^ ions are conversely transported into the nanochannels of the AAM by diffusion or convection. Additionally, precipitates with resultant morphology are obtained with the basis or conditions such like size, shape of the templates. Then, the precipitations with 1-D form decompose by oxidation (Equations (10) and (11)) and are translated into the single crystal CeO_2_ nanowires at 700 °C.

(10) $$2{Ce}^{3+}+3C_{2}O_{4}^{2-}\to Ce_{2}{\left(C_{2}O_{4}\right)}_{3}$$

(11) $$Ce_{2}{\left(C_{2}O_{4}\right)}_{3}+2O_{2}\to 2{CeO}_{2}+6CO_{2}$$

In 2009, Chen *et al.* explained the detailed reaction mechanisms for the formation of ceria nanotubes through the Ce(OH)CO_3_ nanorods using the reaction times [24]. They also extended the reaction with the appropriate amount of basic environment for Ce(OH)CO_3_ nanorods and that dissociated slowly to produce Ce^3+^, OH^−^, and CO_3_^2−^ ions in solution (Equation (12)).

(12) $$Ce(OH)CO_{3}\to {Ce}^{3+}+OH^{-}+{CO_{3}}^{2-}$$

(13) $${Ce}^{3+}+OH^{-}\to {Ce\left(OH\right)}_{3}$$

(14) $${Ce\left(OH\right)}_{3}\overset{O_{2}}{\to }{Ce\left(OH\right)}_{4}$$

(15) $${Ce\left(OH\right)}_{4}\overset{calcination}{\to }{CeO}_{2}$$

The external surface of the Ce(OH)CO_3_ nanorods would lead to the formation of Ce(OH)_3_ (Equation (13)), due to the outward diffusion of Ce^3+^ in the Ce(OH)CO_3_ core being faster than inward diffusion of OH^−^ ions in solution for different ionic radii and would prevent the diffusion of Ce^3+^ and OH^−^ through the shell. In the presence of oxidizing agent, the Ce^3+^ ions present in the Ce(OH)_3_ nanostructures can be converted to a Ce^4+^ complex (Equation (14)). Thus, rod like ceria can be easily formed at the outer wall under the transformation of Ce(OH)_2_^2+^ atoms in the solution. Additionally, Ce(OH)_4_ be dehydrated directly and simultaneously unreacted Ce(OH)CO_3_ completely decomposes during the afterward calcination process in the presence of air to form ceria (Equation (15)). Most of the outer wall is oxidized into ceria and 1-D Ce-NT would be formed. Owing to the early established voids that may lose surface atoms, thus the net inward flow of vacancies converges in a bigger space.

Similar to this approach, there is a template strategy with the presence of CNTs in the case of solvothermal and ultrasonic method [25–31]. During this process, CNTs are first treated by acid or pyridine, and then the surface acidic groups on the nanotubes can adsorb Ce^3+^ and form metal-oxygen bonds [25,30,31]. This phenomenon is observed due to the formation of noncovalent bond on to the surface of CNTs. In the case of the use of pyridine, an aromatic ring can be absorbed on CNTs by the π–π stacking interaction. Therefore, significant amount of water or alkaline solution (NaOH or KOH) may generate OH^−^ around CNTs, and then OH^−^ reacts with Ce^3+^ to produce nanoparticles [25–28,31]. CeO_2_ nanoparticles are absorbed on the CNTs to reduce the surface energy. With increasing time, more and more CeO_2_ nanoparticles absorbed on the CNTs form continuously the coating layer. The CeO_2_ nanoparticles fuse together for a steady structure under the solvothermal condition. Then, the as-prepared composites are heated at 450 °C in an air atmosphere for 30 min to remove CNTs. The diameter of ceria nanotubes was about 40–50 nm. According to the above carbon nanotube assisted results, the possible formation mechanism of the CeO_2_ nanotubes is proposed as shown in Scheme 1. It can be seen that the key steps involved in the formation of the CeO_2_ nanotubes are solvothermal modifications of CNTs and controlled calcinations. Considering the convenience of the procedure, this CNT template-assisted route is promising to extend to the preparation of CeO_2_ necklace-like hollow nanobeads [48]. CeO_2_ hollow nanobeads are 150–200 nm in outer diameter and 40–60 nm in inner diameter.

### 3.2. Non-Surfactant Assisted 1-D CeO_2_ Nanostructure Formation

A pronounced influence of nonsurfactant on the ceria nanostructure has been observed due to simple and economic concerns. Through thermal gravimetric analysis, Huang *et al*. [7] observed that in the basic environment with the presence of air, dehydrated ceria (CeO_2_·*n*H_2_O) nanostructure are deduced to be CeO_2_·1.1H_2_O (Equation (16)) and nanorods exhibit a light yellow color CeO_2_ due to the dehydration of the water (Equation (17)). The reaction steps are similar to those proposed by Dengsong *et al*. [11] in 2005 for the ultrasonication method.

(16) $$4{Ce}^{3+}+12OH^{-}+O_{2}+(4n-6)H_{2}O\to 4({CeO}_{2}.nH_{2}O)$$

(17) $${CeO}_{2}.nH_{2}O\to {CeO}_{2}+nH_{2}O$$

Beside the above reaction, in 2008, Fu *et al*. [43] optimized the nanostructure morphology through changes of electric field and different potential sweep rates. Later on, Pan *et al.* [37] assisted the template free controlled conversion of ceria nanoparticles into 1-D nanostructures with changes in the reaction time and reaction temperature (Scheme 1). They explain the detailed mechanisms with coarsening; also know as Ostwald ripening through the particle size effect. A rate law for this process for the diffusion-limited particle growth in the solid and liquid state is developed by inserting the linearized Gibbs-Thompson equation into Fick’s first law as shown in equation (18). In the equation, *r̄*^3^ is the average particle size at time *t*, ${\bar{r_{0}}}^{3}$ is the average initial particle size, and *k* is the rate constant.

(18) $${\bar{r}}^{3}-{\bar{r_{0}}}^{3}=kt$$

In addition, ideal coarsening kinetics can be determined by equation (19), where, *η* is the viscosity of the solvent, *a* is the solvated ion radius, *V_m_* is the molar volume, *C_r_*_=∞_ is the equilibrium concentration, *N_A_* is Avogadro’s number, and γ is the surface energy.

(19) $$k=\frac{8\gamma V_{m}^{2}C_{r=\infty }}{54\pi \eta aN_{A}}$$

The surface energy for the solid-vapor interface for metal oxides is of the order of 1 J m^−2^. On the other side, electrostatic and chemical interactions at the solid-liquid interface are expected to reduce the surface energy to values in the range 0.1–0.5 Jm^−2^. The difference in solubility, 5.23 × 10^−12^ mol L^−1^ for CeO_2_ and 4.85 × 10^−6^ mol L^−1^ for Ce(OH)_3_, greatly affects the rate constant and thus different nanostructures are obtained. They also observed that the change of precursor significantly affects the formation of the 1-D nanorods due to the effect of Ce^3+^ ions on the reaction mechanism. In addition, the change of rate constant for the oxidation of Ce^3+^ ions to Ce^4+^ ions readily increased the ceria nanotube formation. On the other hand, increases of the rate constant for the deposition of the Ce^3+^ ions on the tips of the nanorod results in the formation of CeO_2_ nanowire rather than nanotube structure and this phenomenon was previously confirmed by Huang *et al*. for the formation of nanorods [7]. Continuous rising of the temperature for the case of surface oxidation modifies the 1-D nanostructure into nanopowder with growth into nanocubes also reported. It is widely accepted that the Kirkendall effect controls experimental conditions for the formation of nanotube structures. Zhou *et al*. [39] carried out comparative experiments with the as-prepared 1-D Ce(OH)_3_ nanorod and H_2_O_2_ to clarify the mechanism for the formation of 1-D Ce-NT with the Kirkendall effect in 2007. They observed that both H_2_O_2_ and partial oxidation of Ce(OH)_3_ are essential for the formation of ceria tubular structure. In this, Ce^3+^ ions present in the Ce(OH)_3_ nanostructure can be converted to a Ce^4+^ complex (Ce(OH)_2_^2+^) by hydroxyl free radicals in the H_2_O_2_ solution, thus Ce(OH)_2_^2+^ transfers into the solution (Equation (20)). As the concentration of the Ce(OH)_2_^2+^ increases, ceria can be formed easily under the Equation (21).

(20) $$2{Ce}^{3+}+3OH^{-}+H_{2}O_{2}\to 2Ce{\left(OH\right)}_{2}^{2+}(aq)$$

(21) $$Ce{\left(OH\right)}_{2}^{2+}(aq)+2OH^{-}\to {CeO}_{2}(s)+2H_{2}O$$

According to the simple hydrothermal method, Tang *et al*. [3] observed the precipitation mode together with the concentration of starting and acidic precipitant environment. This reaction environment played a significantly important role in the formation of the nanowire, confirmed by morphological analyses (Equation (20)). In addition, with the presence of an acid wash, the thickness of the shell and the interior space shrinks slightly, thus forming a hollow tubular nanostructure. Furthermore, Chen *et al*. [41] proposed that Zr^4+^ ions may act as the catalyst to promote the diffuse rate of Ce^3+^/Ce^4+^ ions inside the nanorods. Thus, the metal hydroxide nanorods gradually decompose to form Zr*_x_*Ce_1−_*_x_*O_2_ nanotubes from Zr*_x_*Ce_1−_*_x_*(OH)_3_ rod according to a partial oxidation of Ce^3+^ and differential rates of diffusion between Ce^4+^ and Ce^3+^ ions inside the material, see in Scheme 1.

## 4. Applications of Ceria Nanostructure Materials

The surface oxygen mobility through the lattice of the ceria is allowed to behave as an oxygen buffer and provides ceria an ultimate choice for an application based on the enhancement of the electrochemical phenomenon. In addition, enchantment of the oxygen mobility also varies with particle size and shape of the nanostructures. From the very beginning of nanomaterial research, it has been recognized that the size of the components is alterable. Additionally, shape and structure differences are attractive for several activities. The catalytic activity of many systems has been observed as structurally sensitive. Therefore, several industrial important reactions, including low-temperature CO oxidation, UV absorbing semiconductor materials, partial oxidation of hydrocarbons, hydrogenation of carbon oxides, and wastewater treatment, are exclusively affected by the structure of the nanomaterials.

### 4.1. UV-Vis Absorption

The optical property of absorbance of ceria in the UV region suggests that it can be used as a good candidate for UV absorbing semiconductor materials. To understand the correlation between the band gap energies and the grain size, the morphology of the ceria nanomaterials is important. The UV-Vis absorption spectra of 1-D like bulk nanomaterials, calcined CeO_2_ nanospheres, micro or nano rods, and spindle-like particles are recorded in several research works, represented in Table 1. In the past decade, film type ceria structure has been frequently investigated to understand optical properties in the UV-Vis region. The optical band gap *E*_g_ can be determined from the absorption coefficient according to the solid band theory for a semiconductor and is given by *α*(*hν*)^n^ = constant (*hν* − *E*_g_), where *hυ* is the photo energy, *α* is the absorption coefficient constant is relative to the material. The optical absorption coefficient *α* was calculated from *k* (extinction coefficient) value using *α* = (4*πk*/*λ*), according to the following equation: *α* = (2.303 × 10^3^*A*ρ)/*lc*, where *A* is the absorbance of the sample, ρ is the real density of CeO_2_ (7.28 g cm^−3^), *l* is the path length of the quartz cell (1 cm), and *c* is the concentration of the ceria suspensions. The dependence of the absorption coefficient (*α*(*hν*)) relates to the energy of the incoming photons (*hν*) in the case of materials and *n* is either 2 for a direct transition [E_d_] or 1/2 for an indirect transition with an indirect band gap [E_i_].

The optical direct band gap energy for different 1-D nanostructures was observed by Ho *et al*. [2] through UV-Vis spectroscopy to understand the morphological applications of CeO_2_ nanostructures. Compared to the no-noriented polycrystalline CeO_2_ (*E*_d_ = 3.19 eV), CeO_2_ nanospheres (80–100 nm), microrods (width several 100 nm; length 15 and 20 μm, aspect ratio (AR) = 25–33), and spindle-like structures (width several 100 nm, length 2–4 μm, AR = 4–8) were prepared through polyol process and showed an increase in *E*_d_ by a value exceeding 0.27, 0.43, and 0.17 eV, respectively. According to Zhang *et al*. [49], in the hydrothermal method, spindle-like 1-D nanostructures with particle diameters of about 800 nm and lengths up to five micrometers have a direct bandgap of 3.55 eV. The absorption spectrum indicates that the CeO_2_ spindle is allocated with fairly larger band in different preparation methods for the implication of the particle sizes [2,10,49]. In addition, hydrothermally synthesized prism-like mesocrystal [50] CeO_2_ sample exhibit the direct band gap of 3.02 eV, which is smaller than the value for the bulk CeO_2_ (*E*_g_ = 3.19 eV). It could be ascribed to the coexistence of abundant defects in such prism-like mesocrystal CeO_2_.

Furthermore, Elidrissi, and co workers [51] prepared the CeO_2_ films by using two different kind of solution precursors (cerium chloride and cerium nitrate as the sources of cerium ions) to reveal the optical transmission properties in the spray pyrolysis procedure. The optical properties of CeO_2_ thin films are determined from transmission and reflection measurements in the range of 0.3–2.5 μm. Both films exhibit a transmittance above 80% in the visible and near-infrared region with a sharp absorption edge to approximately 350 nm. The direct band gaps of the films prepared by cerium chloride and cerium nitrate are 3.6 and 3.53 eV, respectively. The difference observed in the band gap resulted from the two different sources of cerium ions. Moreover, Charitidis *et al*. [52] grew the nanostructured CeO_2−x_ films through electron beam evaporation (EBE) and ion beam assisted deposition (IBAD) consisting of grain sizes of 9–28 nm. They kept the nanoscale voids to enhance the surface and quantum-size effect. The optical properties of the CeO_2−x_ sample locate at least ~0.3 eV difference between indirect and direct band gaps. According to Zhang *et al*. [11], 1-D CeO_2_ nanorods were readily synthesized through ultrasonication procedure in the presence of polyethylene glycol. The aspect ratio of the CeO_2_ nanorods were 10 to 15:1, and the length of the nanorods were 50–150 nm in length, with (111) and (220) lattice fringes of 0.31 and 0.19 nm, respectively. In addition, the direct transition band energy (*E*_d_) of 2.90 eV and the indirect band gap energy (*E*_i_) of 2.67 eV for CeO_2_ nanorods were observed. Additionally, the 1-D CeO_2_ nanoparticles synthesized using the microemulsion method were consistent with (*E*_d_ is 3.44 eV for 2.6 nm and 3.38 eV for 4.1 nm, while *E*_i_ is 2.87 eV for 2.6 nm and 2.73 eV for 4.1 nm, respectively) several particle size effects for the band gap analyses [53].

According to Tatar *et al*. [54], the refractive index was determined as 1.8–2.7 in the photon energy interval from 3.5 to 1.25 eV with the optical model. In addition, the optical indirect band gap (*E*_i_) of CeO_2_ nanocrystalline films was calculated as 2.58 eV. The calculated indirect band gap values were lower than the band gap values of other physical vapor-deposited CeO_2_ films (3.15–3.5 eV) [55–57]. However optical band gap values for the spray deposited ceria films are smaller than those of the films prepared by magnetron sputtering at about 800 °C (E_g_ ~3.30 eV) [58]. The differences observed in the band gap values of nano- and micro-crystalline ceria were attributed to the presence of increased oxygen vacancies in the nanocrystalline structure of the ceria, which led to a distortion of the local symmetry. According to Gallage *et al.* [59], ceria films on glass substrates showed high transparency with more than 70% transmittance (85% with respect to the glass substrate) in both visible and infrared regions. The values of the optical band gap for all ceria films are ~3.06–3.08 eV. These values are comparable to the values of ceria films prepared by the sol-gel method at 450 °C (3.03–3.07 eV) [60]. Both sol-gel deposited films and spray deposited films at low temperatures have a smaller grain size with random orientation. Therefore, it can be suggested that the higher concentration of grain boundaries is responsible for the broadening of absorption edge and apparent shift towards the lower energy of the optical band gap. Patsalas *et al*. [61] observed the correlation of the indirect optical band gap with their microstructures and composition of nanocrystalline (grain of 8–40 nm) ceria film prepared by EBE at room temperature and 950 °C. Furthermore, they showed that E_g_ was decreased by increasing Ce^3+^ ion content in EBE film. Several data have revealed that *E*_d_ and *E*_i_ decrease with the increasing size of CeO_2_ nanoparticles owing to the quantum confinement effect [49,52]. In addition, Zhang *et al*. [10] explained that the band gap decreased from 3.95 to 3.86 eV as the reaction temperature increases from 500 to 800 °C. Therefore, reaction temperatures significantly affect the band gap for the 1-D nanostructures. Furthermore, precursor as the sources of cerium ions may affect the band gap properties. Although the detailed reasons are not clear for the increases of the band gap in the nanoparticles, it would be concerned with the size effects [10,11,49–60].

### 4.2. UV-Vis Absorption Shift Phenomenon

Absorption and emission spectra of nanomaterials are readily assisted to ensure an overview of particle size and internal morphology. Theoretically, absorbance band edge shifts towards the shorter wavelength is demonstrated as a blue-shift [2]. On the other side, red-shift is characteristic of the electron–phonon coupling phenomenon [62]. The size of the particle is readily influenced by the quantum confinement consequences [2]. It is well known that decreasing size of materials increases with the electron-phonon-coupling coefficients. In certain systems, electron-phonon coupling could be strong enough to overcome the spatial confinement to determine the energy of excitons. It determines or modifies the effective mass of carriers and the style of carrier scattering by the lattice, leading to a red-shift of the emission band.

The blue-shifting phenomenon in the UV absorption spectra of CeO_2_ nanocrystals has attracted the interest of many researchers in recent years [2,11]. Generally, the absorption of ceria in the UV region originates from the charge-transfer transition between the O 2p and Ce 4f states in O^2−^ and Ce^4+^. This spectral profile indicates that charge-transfer transition of Ce^4+^ overlaps with the 4f^1^ → 5d^1^ transition of Ce^3+^, which overrun the well-known f to f spin-orbit splitting of the Ce 4f state [2,11,52,63]. According to Guo *et al*. [64], ultraviolet blocking materials, CeO_2_ single/multiwall hollow microspheres have strong absorption properties in the ultraviolet range. As the shell thickness increases from 20 to 50 nm, the absorbing boundary of CeO_2_ hollow microspheres is blue-shifted from 450 to 430 nm. A clear blue-shifting of the absorption threshold edge can be observed for the CeO_2_ nanospheres and microrods, contrasting with the bulk powder, due to the decrease of particle sizes [2,49] and can also be affected by temperature [10]. Normally, nanophase crystallinity is expected to lead to blue-shift effects because of quantum confinement. However, the red-shift of the absorption bands of CeO_2_ nanorods, nanoneedles, prism-like mesocrystal and single/multiwall hollow microspheres have specific oriented aggregation of individual nanostructures contributing to the existence of a large number of defects [1,50,62]. In addition, the red-shift effect observed in the nanocrystalline ceria would be explained by the formation of localized states within the band gap owing to oxygen vacancies and increase Ce^3+^ ion concentration. This phenomenon is due to the shift of absorbance band shift towards the longer wavelength [50,52].

### 4.3. Carbon-monoxide Oxidation Phenomenon

In recent years, oxidation catalysts have received considerable attention because of their potential role in the environmentally important fuel cell technologies. As an important component in catalysts, ceria promotes high oxygen storage capacity (OSC) and high oxygen ion conductivity. Several morphological structures of CeO_2_ such as nanorod, nano-sponge single or multiwall, hollow structure, mesoporous, spindle *etc*., have been investigated widely for the selective oxidation of mainly carbonmonoxide, nitrogen oxides, sulfur oxide, and so on, due to OSC of ceria. In addition, surface area, structural defects, and oxygen vacancy have a positive effect on CO oxidation [2]. The formation of oxygen vacancy can be expressed by the following equation (22):

(22) $$4Ce_{\left(s\right)}^{4+}+O_{\left(s\right)}^{2-}\to 4{Ce}^{4+}+2e^{-}/V_{\left(o,s\right)}+\frac{1}{2}O_{2}\to 2{Ce}^{4+},2Ce_{\left(s\right)}^{3+}+V_{\left(o,s\right)}+\frac{1}{2}O_{2}$$

where *V*_(O,s)_ represents an empty position (anion-vacant site) originating from the removal of O^2−^ from the lattice. Charge balance is maintained by the reduction of two cerium cations from +4 to +3. The radius of the Ce^3+^ ion (1.14 Å) is larger than that of Ce^4+^ (0.97Å) and hence the lattice expansion is a consequence of the reduction of Ce^4+^ ions to Ce^3+^. There is a gradual decrease in the concentration of oxygen vacancies extended from the surface to the bulk. Such gradient enables the outward diffusion of lattice oxygen to the surface. Therefore, the reduction of Ce^4+^ to Ce^3+^ by oxygen ion leads to the generation of surface oxygen vacancy. These oxygen vacancies can act as promoting sites for NO and CO conversion [62–70].

The catalytic performance of the 1-D CeO_2_ nanomaterials is affected by the structure and surface area as shown in Table 2. Zhang *et al*. compared the two different kinds of 1-D nanomaterials to exhibit CO oxidation, where they derived that CeO_2_ single/multiwall hollow microspheres may provide CO total conversion at 230 °C and for bulk CeO_2_ at 500 °C [62]. Hollow microspheres afford more available oxygen and oxygen deficiency for CO oxidation [48,62,66]. In addition, high catalytic activity on CO oxidation obtained for CeO_2_ single/multiwall hollow microspheres was consistent with similar activity at 240 °C for *T*_100_ in the second and the third runs, which revealed its excellent thermal stability and recycling performance [62]. Similar tendency of the CO oxidation was followed for the hollow nanobeads and hollow nanocubes [48,67]. CNT templates in the CeO_2_ hollow nanobeads may be formed of CeO_2−_*_x_*C and thus increase the catalytic activity [48]. According to Chen *et al*. [68], the CO conversion of CeO_2_ hollow nanocubes is 56% and almost 3.5 times higher than that of the CeO_2_ powder at 270 °C. They explained that the interconnected hollow structure enables better contact with the gas molecule owing to the existence of interior spaces and penetrable shells, therefore exhibit better performance. The stability and recycling performance of CeO_2_ catalysts are important factors for the practical applications.

According to TEM analyses, they demonstrated that the hollow structure does not collapse at high temperature (300 °C) and the catalytic operation was conducted after the reactor cooled down to room temperature, which demonstrated its excellent stability and recycling performance [66,68]. On the other hand, the overall catalytic activity and the BET specific surface area are affected by the preparation method of the catalyst [5,53]. Masui *et al*. [53] reported that the CeO_2_/Al_2_O_3_ catalyst prepared by the microemulsion method shows higher activity for carbon monoxide oxidation, despite the fact that the CeO_2_/Al_2_O_3_ catalyst surface area is as low as that prepared by the co-precipitation method. Pan and coworkers [5] explained that nanomaterials consisting of similar BET specific area greatly influence the crystal surface to represent the catalytic activity. They also observed that CeO_2_ nanorods, nanoplate, and nanotubes exhibit higher BET surface area: 52.5, 37.2, and 80.1 m^2^g^−1^ respectively, as well nanoplates, consistent with higher crystal surface (100), and that contributes to create enormous oxygen vacancies, thus favors the higher catalytic performance.

Regarding the several types of nanostructures such as spindle, rod *etc*., the effect of carbon monoxide oxidization of CeO_2_ was revealed by Ho *et al*. [2]. The spindle-like sample shows the highest CO conversion rates 0.861 μmol g^−1^ s^−1^, which is almost 4.5 times that of CeO_2_ particles (as referred), 0.189 μmol g^−1^ s^−1^. According to XRD analysis, they observed the order of the lattice cell volume was strongly related to the degree of Ce^4+^ reduction and the extent of oxygen vacancy. Interestingly, the surface area and pore volume of the samples significantly increased after calcinations and affected the CO oxidation. The same result for the effects of surface area was also demonstrated for the ceria nanorod and sponge nanorod [6,45]. Zhou *et al*. [6] attained that CeO_2_ nanorods are three-times more active than CeO_2_ nanoparticles for CO oxidation and found that the *T*_100_ (the temperature at which the CO conversion is 100%) for the CeO_2_ nanorods and CeO_2_ nanoparticles catalysts approach to 275 and 300 °C, respectively. In addition, using CeO_2_ sponge-like rods as a catalyst, the *T*_100_ is only 205 °C, which shows that catalytic property of CeO_2_ sponge-like rods has an advantage over that of CeO_2_ nanorods and CeO_2_ nanoparticles [45]. The sponge nanorod may provide a larger percentage of atoms onto the surface and would create structural defects and generate pronounced oxygen vacancies than nanorod or nanoparticles [6,45,68]. Similar tendency is also observed for the case of ceria nanotube [26], therefore it could provide the three times higher catalytical activity than bulk ceria and ceria nanoparticle.

Recently, Pan *et al*. [37] explained that 1-D ceria nanorods synthesized at low temperature with enough aging time can possess a large BET specific area and thus provide a perfect crystalline form and have high performance for CO oxidation. The physical and chemical properties of ceria can be tuned by doping with different metals to obtain low-temperature reducibility (Au, Cu, Pr and Sn). Metallic doping with tetravalent cations, (such as Zr and Hf) onto the ceria nanostructures may enhance the OSC and consequently archive high ionic conductivities with trivalent cations (such as La, Sm, Gd, and Y) [69–71]. Sunder *et al*. [69] observed that the catalytic activity of the CO oxidation with Cu-CeO nanocomposite can significantly increase due to the addition of CuO. Similar research was also conducted by Sun *et al*. [70], who observed that the quickly accelerated CO conversion starts below 120 °C, and complete CO oxidation is achieved at about 220 °C over the catalysts containing more than 10 wt% CuO on to the 3-D flower-type CeO_2_ nanomaterials. The performance of the flowerlike CeO_2_ microspheres loaded with 20 wt% CuO became worse and the 15 wt% CuO sample had the best catalytic activity for CO oxidation. The activity may be affected by higher CuO content or the surface-volume ratio of the catalyst [69,70].

The role of the support and the oxygen supply for the catalytic reaction remain controversial. Although it is accepted that factors, such as gold particle size, synthesis method, pretreatment conditions, and support, influence the reactivity of the supported gold catalysts, the nature of the active sites and the reaction mechanism for CO oxidation are still subjects of debate. According to Raman spectroscopic analyses, Guzman *et al*. [71] indicated that nanocrystalline CeO_2_, in the presence of gold catalyst, supplies reactive oxygen in the form of surface η^1^ superoxide species and peroxide adspecies. The conventionally precipitated CeO_2_ tends to stabilize O_2_^δ−^ (0 < δ < 1) adspecies and molecular O_2_ on to the surface. Thus, both cationic and metallic gold are attributed in nanocrystalline CeO_2_ to accelerate CO oxidation at low temperatures. The formation of the surface chemisorbed oxygen species can be facilitated by defects in the catalyst structure. Therefore, Sun *et al*. [70] demonstrated that 2.77 wt% Au-loaded flower-like CeO_2_ microsphere catalysts highly active with CO gas conversion into CO_2_ above 80% at room temperature and T_100_ is observed at 130 °C. On the other hand, around 81% CO conversion is achieved at 220 °C for Au/CeO_2_ nanorods as a catalyst, while only 20–22% CO conversion is obtained at the same temperature for pure CeO_2_ nanorods and nanoparticles as a catalyst [7]. The catalytic activities of Au/CeO_2_ flower, nanorods, and nanoparticles are much higher than that of pure CeO_2_ nanorods and nanoparticles, consequently [7,70].

## 5. Conclusions

Ceria nanomaterials have received attraction in the past decade due to their effective applications in the fields of environmental protection and in semiconductor industries. One-dimensional ceria nanostructures have reached such potential owing to their size, shapes and crystallographic behaviors. Due to their preparation procedure and preparation mechanism, different 1-D ceria nanostructures can be accomplished. To improve the properties of the ceria nanomaterials in terms of environmental and other issues, an enormous amount of reaction mechanisms and preparation procedures have been developed. So far, correlations between the details of ceria nanomaterial preparation and the mechanisms of the 1-D nanomaterial have not been established. Therefore, an overview of several 1-D ceria nanomaterials like nanorod, nanowire/nanofiber, nanotube *etc*., and the preparation mechanisms and applications are provided in the present work, and should facilitate the choice of the right type of ceria for a specific application, as well as to provide a better understanding for designing new ceriabased materials with the desired properties.

## Figures and Tables

**Table 1:** Details of the ceria nanomaterials UV-Vis absorption analyses.

References	Preparation Procedure	Sample	Band Gap a(eV)	
			E_d_	E_i_
e[2]	Polyol	Polycrystalline CeO_2_	3.19	N.A.
		CeO_2_ nanospheres (80–100 nm),	3.46	
		Microrods (d WD several100 nm; d L 15 to 20 μm, d AR 25 to 33),	3.62	
		Spindle-like (d WD several 100 nm, d L 2 to 4 μm, d AR 4-8)	3.36	
[49]	Hydrothermal	Spindle like (d WD 800 nm and d L 5 μm)	3.55	N.A.
[50]	Hydrothermal	CeO_2_ prism-like mesocrystal Bulk CeO_2_	3.02 3.19	N.A.
[51]	Spray pyrolysis	CeO_2_ films (cerium chloride) (cerium nitrate)	3.6 3.53	N.A.
[52]	Electron beam evaporation; Ion beam assisted deposition	Nanostructured CeO_2−x_	3.48	3.18
e[11]	Ultrasonication	CeO_2_ nanorods (d AR 10 to 15:1, d L 50–150 nm)	2.9	2.67
[53]	Microemulsion	Ceria ultrafine nanostructure	3.44 2.6	2.87 2.73
[54]	Pulsed electron beam	CeO_2_ nanocrystalline films	N.A.	2.58
[56–58]	Physical vapor-deposited	CeO_2_ films	N.A.	3.15–3.5
[59]	Spray deposition	CeO_2_ films	N.A.	3.06–3.08
[60]	Sol-gel method	CeO_2_ films	N.A.	3.03–3.07

Notes:

a According to the solid band theory for a semiconductor (*hν*)^n^ = constant(*hν* – *E*_g_), where *hυ* is the photo energy, *α* is the absorption coefficient, constant is relative to the material, *E_g_* is the band gap;

b E_d_: Band gap energy for direct transitions in where n = 2; E_i_: Band gap energy for indirect transitions in where n = 1/2;

d AR = aspect ratio; L = length; N.A. = not available; WD = width;

e Surfactant method.

**Table 2:** Carbon-monoxide oxidation effect on several ceria nanostructures.

References	Sample	T_50_ [°C]	T_100_ [°C]	BET [m^2^g^−1^]	Remarks
[54]	CeO_2_/Al_2_O_3_	270	N.A.^d^	165	Microemulsion method provides higher catalytical activity
	Microemulsion	N.A.	N.A.	73	
	CeO_2_/Al_2_O_3_	320	N.A.	167	
	Coprecipitation	N.A.	N.A.	73	
[62]	CeO_2_/single multiwall	210	230	44.9	In the second and third run, provides 100% conversion at 240 °C.

[67]	CeO_2_ hollow	265	N.A.	N.A.	Similar conversion provided at the second run.
	Commercial	>300	N.A.	N.A.	
[69]	Mesoporous CeO_2_ with	N.A.	220	N.A.	Higher content of the CuO may alter the surface to volume ratio of the catalyst and affect the gas transfer.
[70]	CuO	N.A.	N.A.	N.A.	
	Bulk CeO_2_	N.A.	500	141	
	Nano CeO_2_(NC)	435	N.A.	N.A.	
	2%Cu-NC	166	N.A.	107	
	10%Cu-NC	148	N.A.	131	
	20%Cu-NC	150	N.A.	118		
[26]	Bulk CeO_2_	>300	N.A.	5.67	Ceria nanotubes are more active than the ceria nanoparticles and bulk ceria due to large surface area.
	CeO_2_ nanoparticle	298	N.A.	30.33	
	CeO_2_ nanotube	205	275	83.15	
e[5]	Nanoplate	215	>300	37.2	Crystal plane (100) greatly affects the oxidation.
	Nanorod	273	>340	52.5	
	Nanotube	264	>325	80.1	
e[2]	CeO_2_-nanoparticle	295	380	N.A.	BET surface area increases after the calcination at 400 °C and that may influence the conversion.
	Spherical	284	315	40.3	
	Rods	265	315	67.8	
	Spindle	250	300	67.4	
[6]	CeO_2_ nanorod	N.A.	275	50.1	N.A.
[45]	CeO_2_ nanoparticle	N.A.	300	62.4	
	CeO_2_ sponge rod	190	205	N.A.	
[70]	Au/CeO_2_ nanorod	N.A.	>220	N.A.	Au-supported nanoparticle provides better conversion due to the thermal stability.
	CeO_2_ nano particle	>220	N.A.	N.A.	
	CeO_2_ nanorod	>220	N.A.	N.A.	
	Au/CeO_2_ nano particle	N.A.	160	N.A.	
[48]	Ceria nanobead	240	300	87.5	CNT templates in the CeO_2_ hollow nanobeads may be formed from CeO_2_^− x^C.
	Ceria nanoparticle	>300	N.A.	5.7	
f[37]	Nanorod^a^	290	N.A.	128.2	Possesses enough aging time to increase BET surface area and consequently affect oxidation process.
	Nanorod^b^	224	N.A.	115.9	
	Nanoparticle	305	N.A.	105.1	
	Nanowire	245	N.A.	79.8	
	Nanotube	223	N.A.	98.2	
	Nanocube^c^	315	N.A.	3.5	

Notes:

a CeO_2_ nanorods synthesized at 20 °C for 24 h;

b CeO_2_ nanorods synthesized at 20 °C for 9 d;

c CeO_2_ nanocubes synthesized at 180 °C for 24h;

d “N.A.” denotes “not available”;

e Surfactant method;

f Nonsurfactant method.
